# A single session of mindfulness meditation may acutely enhance cognitive performance regardless of meditation experience

**DOI:** 10.1371/journal.pone.0282188

**Published:** 2023-03-15

**Authors:** Rita Sleimen-Malkoun, Louise Devillers-Réolon, Jean-Jacques Temprado

**Affiliations:** Aix Marseille Univ, CNRS, ISM, Marseille, France; Liverpool John Moores University, UNITED KINGDOM

## Abstract

The present study investigated acute cognitive effects of mindfulness meditation (MM) compared to an active control intervention in meditators (n = 22) and novices (n = 20) using a within-subject design. We analyzed reaction times in a digitized Stroop task at baseline, after a 10-minute MM session with a fundamental breathing exercise, and after a 10-minute attentive listening intervention. Interventions order was randomized and a 10 min delay was respected before testing. Relative to baseline, meditators and novices showed faster reaction times after both interventions, but more so after MM for the congruent and incongruent Stroop task conditions that are associated with attention, inhibition and cognitive flexibility. Although the two interventions showed cognitive effects independent of previous meditation experience, MM appeared to induce larger benefits. Our findings are encouraging and support MM’s potential as a means to enhance cognitive performance on the short-term without the need of any previous practice.

## Introduction

In the last 15 years, mindfulness meditation (MM) has become a legitimate area of research in behavioral and brain sciences [1]. This secular mental practice originating from Buddhism consists in training one’s attention to be fully drawn to the immediate moment with a sense of curiosity, openness, and acceptance [2, 3]. It aims at developing the trait of mindfulness, that is, a self-awareness of the present experience, including one’s thoughts, feelings, and sensations, without any judgment, filter, or expectations [2, 3].

In the field of cognitive psychology, the trait of mindfulness is described as a metacognitive skill supported by two main components, that is, the orientation to experience and the self-regulation of attention [3, 4]. The latter one presumably involves three cognitive functions: attention, cognitive flexibility, and cognitive inhibition [3]. Attention is necessary to orient, maintain and supervise one’s focus that should be fully drawn to the present moment, or a specific stimulus (e.g., the breath). Cognitive flexibility plays a role in bringing back one’s focus to the present moment when distracted. Cognitive inhibition enables the suppression of elaborative processing of thoughts, feelings, and sensations, as well as irrelevant stimuli.

In the current scientific literature several studies support the benefits of long-term MM practice on cognitive functions, especially those involved in mindfulness, namely attention (e.g., [5, 6]), cognitive flexibility (e.g., [5, 7]), and inhibition (e.g., [7, 8]). However, inconsistent findings have also been reported by other studies, in which MM-related cognitive improvements were not found (e.g., [9] for attention, cognitive flexibility and inhibition [7], for cognitive flexibility). Short-term MM practice is under-investigated, with similarly discrepant results reported regarding their cognitive effects. Specifically, amongst the few existing studies, some reported cognitive benefits ([10] for attention; [10, 11] for inhibition), while others found no effects ([12] for inhibition and cognitive flexibility; [13, 14] for attention and inhibition).

These conflicting results regarding MM’s cognitive benefits (for reviews see [15–17]) could be due to the wide heterogeneity of the methods used, such as: the experimental design (cross-sectional vs. longitudinal), the participants’ characteristics (age, healthy vs. clinical population), the presence and type of the control group (active vs. passive), the choice and implementation of the cognitive tests, and the length and content of the MM intervention. We contend that in addition to these methodological disparities, there are several confounding factors that are often neglected in most studies, which prevents reaching a consensus regarding the presence and the determinants of MM cognitive benefits.

A first possible confounding factor could be the familiarity with MM practice, which is not systematically accounted for in the literature. For instance, the studies investigating acute cognitive effects of MM provide no comparison between meditators and meditation-naïve participants, to whom we refer here as novices. A second possible confounding factor could be the individual-dependant response to MM, i.e., individuals being more or less disposed to benefit from this practice. The distinction between responders and non-responders to interventions has already been successfully applied in the context of physical exercise [18]. In the context of MM, it is supported, inter alia, by studies showing that personality traits moderate the cognitive [19], psychological [20] and physiological [21] benefits of MM practice. A third possible cofounding factor could be the difficulty to enter into a meditative state, i.e., the individual receptivity to the proposed MM intervention. In the current literature, this is often not measured at all (e.g., [22]), or through self-reported questionnaires, which are either validated and evaluating the mindful state reached by the participants during the MM session (e.g., [9]), or custom-made and evaluating the effort put by the participant’s to follow the intervention (e.g., [11]). Such measures can under or over-estimate the individual intervention receptivity as they depend on the participants’ ability to interrogate their mind and to their willingness to follow the instructions.

We contend that studying the acute effects of short-term practice while taking into account the above-mentioned cofounding factors would help in clarifying MM’s cognitive effects and mechanisms of action, while guiding the investigation of longer-term cognitive benefits. Accordingly, the present study, we compared the acute cognitive effects of a guided MM session to that of an active control intervention, isolating thereby the specific effects of MM. Participants with (meditators) and without (novices) previous MM experience were enrolled in the study to determine whether the familiarity with mindfulness practice could affect the observed outcomes. Additionally, to limit the bias related to the individual-dependant response, we used a within subject design in which all participants followed both interventions in a randomized order. Furthermore, we used both objective (heart rate) and subjective (self-reported questionnaire) measures to evaluate whether participants managed to follow the MM intervention. A self-reported questionnaire was implemented to measure the extent to which the participant succeeded in following the proposed mindfulness session. The participants’ heart rate (HR) was measured at rest and during both the MM and the control interventions. Although there is so far no absolute robust objective measure, the literature appears to be supportive of HR as a helpful indicator of meditation effects. Indeed, it has been shown to be significantly lowered during meditation when comparing meditators to a passive control group [23], or within subject meditation to a passive intervention [24].

To assess the cognitive functions of interest, we used a computerized version of the Stroop word-color task [25] that is a broad spectrum cognitive test [26] with a good test-retest reliability [27]. The Stroop task involves mainly cognitive flexibility and attention allocation to select relevant information, as well as inhibition to avoid distraction [28–30]. It has been previously used in many MM studies to assess attention (e.g., [31]), and/or cognitive flexibility (e.g., [5]), and/or inhibition (e.g., [9, 22]).

We expected to observe an improved cognitive performance after the MM session, reflected by faster reaction times in the Stroop task, compared to baseline and to after the control intervention. We also expected to observe a possible interaction between the intervention effect and the familiarity with the practice, which could be explained through the indicators of the engagement in the MM session (self-reported questionnaire and HR).

## Method

### Participants

The sample size was chosen based on G*Power analysis. It indicated that for repeated measure ANOVA (2 groups, 3 tests) at least 28 participants would be required for a medium effect size (f = 0.25, α = 0.05, 1-β = 0.080, ρ = 0.5, [32]). More participants than planned were included in order to prevent any drop-out or difficulties to follow the proposed interventions or even problems with data acquisition.

Forty-two healthy adults volunteered to participate in the study. None of them had previous or current neurological or psychiatric disorders, recent or current musculoskeletal problems of the dominant upper-limb, ongoing psychopharmacological medication, uncorrected vision, an addiction to illegal substances or alcoholism. They were recruited through advertisements in a newspaper, a sports club and via instructors of MM. Twenty-two participants were regular meditators (mean age ± SD: 49.23 ± 13.19 years, 14 women) and twenty had no prior experience in meditation practice, which we called novices (41.50 ± 13.28 years, 11 women). In the meditators’ group, participants practiced MM, or a form of meditation with mindfulness exercises, for at least three-times a week (mean frequency ± SD: 5.53 ± 1.68 sessions per week), since minimally four months (mean experience: 63.5 months, range: 5–250 months). Accordingly, the meditators and novices groups differed regarding their experience in meditation, but were not statistically significantly different regarding sex (chi-square test: χ^2^(1, 42) = 0.32, *p* = 0.57) and age (t-test: t(40) = 1.89, *p* = 0.066, *d* = 0.584).

Prior to their enrolment, all participants were given detailed written information about the study, without stating its precise objective or the underlying hypotheses. They all gave their written informed consent to the experimental procedure that agreed with the Declaration of Helsinki and was approved by the ethics committee of Aix-Marseille University.

### Design

Prior to the experimental session, participants were requested: (i) two hours before, to abstain from smoking and drinking alcohol, tea, coffee or energy drinks, as well as avoid copious meals (fatty foods, animal proteins), (ii) the D-day, to not practice meditation or sport activities.

The experiment was conducted in a quiet room. After being greeted, the participants had an information phase, during which they got familiarized with the experimental room, as well as the used procedure, methods and task.

After signing the consent form, the participants’ resting-state heart rate was recorded for 5 minutes, and then their cognitive performance was tested with the Stroop task at baseline (T_0_). They followed afterwards, in a randomized order, either a control intervention, so-called attentive listening (AL), or a guided mindfulness meditation intervention (MM). During both interventions, their heart rate was recorded. Following both interventions, their cognitive performance was tested again (T_AL_ after AL and T_MM_ after MM). A ten minutes delay post-intervention was respected in order to optimize the chances of observing acute effects [22]. During the ten-minute break after the AL, participants were asked to summarize the content of the recording they just listened to. During the break following the MM, participants filled out a self-assessment questionnaire (SAQ) evaluating their engagement in the meditation session. During each break, participants were invited to get a drink, get up and move around the room.

During the resting state, the two interventions (MM and AL) and the three testing phases (T_0_, T_AL_, and T_MM_), participants were seated comfortably in a height-adjustable chair with their back straight leaning against the seatback, their feet on the floor (or a footrest when necessary), and their hands resting on their thighs (excepting during the Stroop task in which they responded with their dominant hand).

### Interventions

During both interventions, a ten-minute audio recording was played through Bluetooth sports earphones (KeyOuest, model K0014998, France), with the audio volume adjusted to the participants’ convenience. Both recordings featured the same voice with the same slow pace of speech, and similar breaks in their occurrence time and duration.

The AL and MM recordings were conceived and provided by an experienced meditation instructor who trained in several meditative practices including Mindfulness Based Stress Reduction (MBSR) and Vipassana. He is the founder of a guided-meditation mobile application.

The MM recording consisted in a guided fundamental breathing exercise inspired by fundamental Mindfulness Based Stress Reduction programs. In this type of exercise, attention is focused on the sensation of the breath to avoid distractions and redirect awareness in a non-reactive and non-elaborative manner to the present moment [3, 33–36]. Participants were asked to listen attentively to the recording and follow, as closely as possible, the given guiding instructions.

The AL recording offered an informative general introduction to the history, the origins and the philosophy of MM, without inviting to practice it. It required a selective and sustained attention but involved no specific instructions. Participants were requested to remain focused on the recording and to avoid distractions, falling asleep or meditating.

In both interventions, the participants were not allowed to talk, and were required to keep their eyes closed and stay as still as possible.

### Questionnaires

#### The verbal exchange following the AL

The participants’ engagement in the AL was assessed by asking the participants to verbally summarize the content of the recording and express their opinion regarding the clarity of the given information. According to the coherence of their response, the experimenter evaluated their engagement as satisfactory, average or unsatisfactory.

#### The self-assessment questionnaire following the MM

To evaluate how each participant experienced the MM and how well they think they followed the given instructions, we asked them to fill a customized self-assessment questionnaire (SAQ). The SAQ was inspired by Wenk-Sormaz [11] and was based on the common assessment practice in the field of MM. The participants had to report on a scale ranging from 1 (“not at all”) to 10 (“totally”) to which extent they were following the instructions (S1), focusing on their breath (S2), and focusing on their feelings (S3). They were also asked to estimate the percentage of time they spent observing their breath during the meditation exercise (S4). The internal consistency of the four items (S1-4) was found to be satisfactory (Cronbach’s α = 0.8), and were thus used to derive a general score over 10 as follows (Eq 1), with higher scores indicating a better auto-reported success in following the instructions during the MM.


$$SAQ=\frac{S1+S2+S3+\frac{S4}{10}}{4}$$ (Eq 1)

### Heart rate

Heart rate (HR) was used as an objective biomarker of meditation ability to indirectly assess how well the participants followed the meditation exercise. The time (in seconds) between two heart beats (NN interval) was recorded at rest at the beginning of the experimental session and during both interventions using the heart rate monitor POLAR RS 800CX RUN (Polar Electro Öy, Kempele, Finland). During the resting state, participants were asked to close their eyes, remain still, and let their mind wander without falling asleep or meditating.

The mean heart rate (HR) in beats per minute (bpm) was computed as in Eq 2:

$$HR=\frac{Nb(NN)}{\sum \left(NN\right)}$$ (Eq 2)

with *Nb*(*NN*) representing the number of NN and Σ(*NN*) representing the total duration in minutes of all NN.

Before computing HR, raw NN data were preprocessed by excluding: i) NN values that were higher than 1.5 seconds or lower than 0.4 seconds (considered as artefacts), ii) NN values that were below or above 3×SD of the participant’s mean (considered as outliers), and iii) equal adjacent NN values associated to the non-detection of the cardiac activity by the device. Time-series that lost more than third of their data points after preprocessing were excluded. Overall, this led to the loss of eight observations (amongst 129 observations in total) in four meditators: three at resting-state, three during AL and two during MM. Those four participants were thus not included in data analysis.

#### Stroop task

To assess cognitive abilities, we used a computerized version of the Stroop word-color task [25] implemented in MATLAB R2015b (MathWorks, Natick, MA, USA). The Stroop task consists in identifying the font color in which a word is written, without paying attention to the semantic meaning of the word. Two categories of words were displayed on a screen with a black background in four different colors (red, blue, green or yellow): color words (‘rouge’—meaning red-, ‘bleu’ -meaning blue-, ‘vert’ -meaning green-, ‘jaune’ -meaning yellow-) and neutral words (‘jambe’ -meaning leg-, ‘bras’ -meaning arm-, ‘pied’ -meaning foot-, ‘main’ -meaning hand-). This created three conditions: (i) the congruent condition with the font color matching the semantics of the displayed color word (e.g., the word ‘rouge’ in red), (ii) the incongruent condition with the font color not matching the semantics of the displayed color word (e.g., the word ‘rouge’ in green), and (iii) the neutral condition with the font color not being related to the semantics of the displayed neutral word (e.g., the word ‘pied’ in red). Participants were requested to identify the font color of the displayed word by pressing the corresponding key as quickly as possible on a modified keyboard of only four adjacent keys (“R” for rouge -red-, “B” for bleu -blue-, “V” for vert -green-, “J” for jaune -yellow-) using only one finger (always the same) of their dominant hand. The displayed word remained on the screen until the participant pressed a key, and the next word immediately appeared. Seventy-five words were presented in one mixed and randomized block: twenty-five congruent words, twenty-five incongruent words, and twenty-five neutral words.

To ensure that participants had no trouble with performing the task, before the first test at T_0_, they were asked to identify the four different colors on the screen orally, and they completed twelve Stroop familiarization trials (four congruent, four incongruent and four neutral words). We contend that the familiarization phase and the full randomization of the displayed trials reduced the risk of observing a repetition effect due to the within subject design.

At the three tests (T_0_, T_AL_ and T_MM_), for each condition and each participant, we calculated the mean number of errors. Then, only for successful trials, we measured the reaction time (RT) in milliseconds (RT_C_ for congruent words, RT_I_ for incongruent words, RT_N_ for neutral words). Lower RT means faster response.

Before computing mean RT we verified that no value exceeded 3 seconds or were below 200 milliseconds, and that none of the individual values were above or below 3×SD of the participant’s mean for the respective condition and test.

### Data processing and statistical analysis

Data were processed with MATLAB R2018b (MathWorks, Natick, MA, USA) and Microsoft Excel 2010 (Microsoft Corporation, Impressa systems, Santa Rosa, California, USA), and statistically analyzed using the STATISTICA software (version 12, StatSoft Inc., Tulsa, OK, USA).

The scores of SAQ are reported as means with standard deviation (M ± SD). HR and Stroop task variables (number of errors and RT) are reported as means with within-subject correlation-adjusted error bars (M ± CI), with CI representing the 95% normalized confidence interval [37, 38]. To study group differences (meditators vs. novices) in the scores of SAQ, we used independent sample t-tests. For HR, we used a two-ways repeated measures ANOVA with intervention (3: resting-state, MM, AL) × group (2: novices, meditators). For the number of errors and RT in Stroop task, we used a three-ways repeated measures ANOVA with condition (3: congruent, neutral, incongruent) × test (3: T_0_, T_AL_, T_MM_) × group (2: novices, meditators).

Normality (Shapiro-Wilk test) was verified (and met) before conducting t-tests. For the ANOVA, when the assumption of sphericity (Mauchly test) was violated, the Greenhouse-Geisser correction was applied, and the corrected degrees of freedom and p value (*p*_cor_) were reported. The level of significance was set to 5% (*p* < 0.05). Effect sizes are reported as Cohen’s d effect size for independent sample t-tests, and partial omega squared (ω^2^) for ANOVAs. If effects were significant, Fisher’s LSD post-hoc comparisons were conducted to identify significant pairwise differences. This test was shown to restrict family-wise error rate to alpha up to 3 groups as in this study [39]. For the sake of brevity, only values of significant effects are reported in the text. The complete results are provided in Tables 1 and 2.

**Table 1:** ANOVA results of HR.

	F	df	p	Ω²
Group	1.10	1,36	0.30	0.001
***Intervention***	***10*.*99***	***1*.*32*,*58*.*35***	***<0*.*001***	***0*.*010***
Intervention×Group	1.04	2,72	0.36	<0.001

Significant results are in bold italic.

**Table 2:** ANOVA results of Stroop task variables.

	F	df	p	Ω²
**Errors**				
Group	1.10	1,40	0.30	0.001
***Condition***	***16*.*18***	***1*.*65*,*65*.*87***	***<0*.*001***	***0*.*167***
Condition*Group	1.56	2,80	0.22	0.007
Test	2.09	2,80	0.13	0.012
Test*Group	1.38	2,80	0.26	0.004
Condition*Test	2.17	4,160	0.07	0.019
Condition*Test*Group	1.11	4,160	0.35	0.002
**RT**				
Group	1.67	1,40	0.20	0.008
***Condition***	***100*.*47***	***1*.*6*,*64***	***<0*.*001***	***0*.*151***
Condition*Group	1.26	2,80	0.29	<0.001
***Test***	***52*.*92***	***1*.*4*,*59*.*12***	***<0*.*001***	***0*.*137***
Test*Group	0.62	2,80	0.54	<0.001
Condition*Test	***5*.*83***	***4*,*160***	***<0*.*001***	***0*.*007***
Condition*Test*Group	1.60	4,160	0.18	<0.001

Significant results are in bold italic.

## Results

### Questionnaires

Statistical analysis of the SAQ revealed a significant effect of group (respectively, *t*(40) = 2.49, *p* = 0.017, *d* = 0.768). Meditators reported being more engaged in the MM session (8.43 ± 0.94 /10) than novices (7.60 ± 1.23 /10). The grand average score (novices and meditators combined) of the SAQ was 8.04 ± 1.15 /10. Regarding the verbal exchange relating to the AL intervention, all participants of both groups provided a coherent summary relative to the content of the recording. The experimenter deemed oral feedbacks of all participants as satisfactory.

### Heart rate

**Fig 1:**
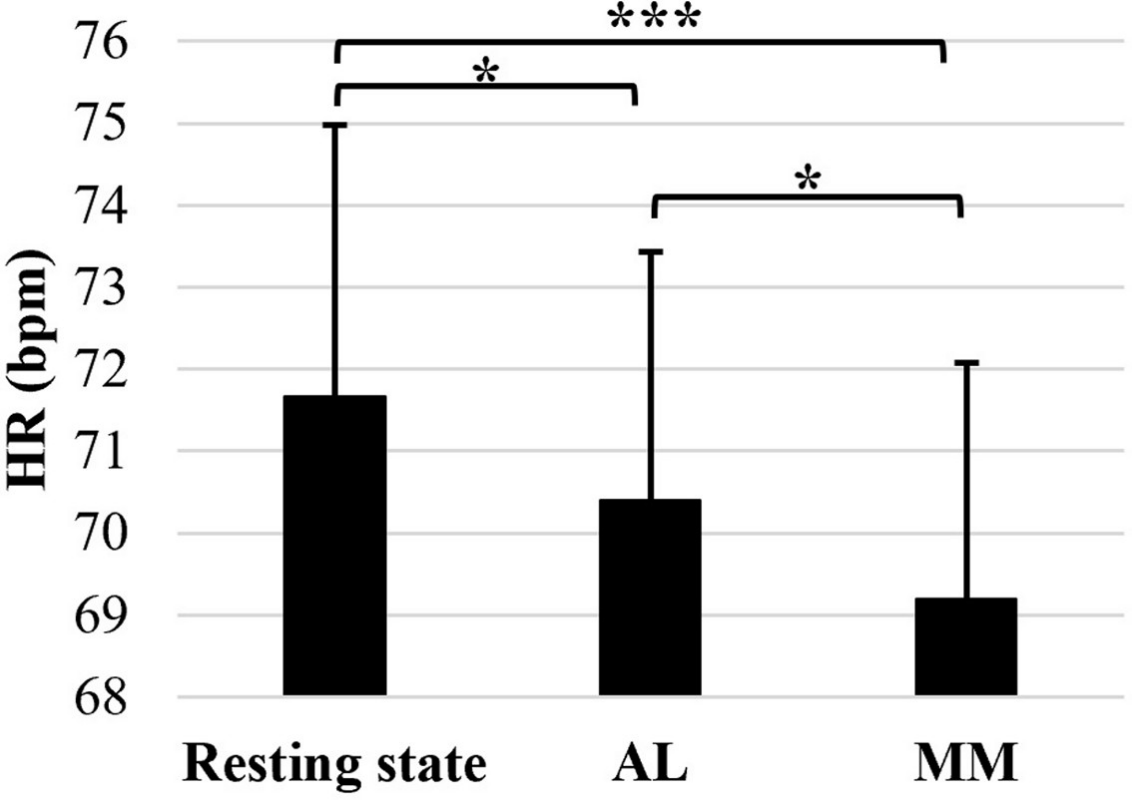
Mean heart rate (HR). Mean HR (in bpm) observed for all participants (meditators and novices combined) during the baseline resting state, the active control (AL) and the meditation (MM) interventions. Error bars represent the normalized 95% confidence interval. *p ≤ 0.05 and ***p ≤ 0.001.

For the mean HR (S1 Fig), the ANOVA’s results (Table 1) revealed a significant effect of intervention (*F*(1.62,58.35) = 10.99, *p*_*cor*_ < 0.001, *Ω*² = 0.010). Post-hoc comparisons showed that, irrespective of their group, the participants’ HR was significantly higher at resting-state (71.66 ± 3.32 bpm) than during AL (70.39 ± 3.03 bpm, *p* = 0.021) and during MM (69.18 ± 2.89 bpm, *p<* 0.001), and lower during MM compared to during AL (*p* = 0.027) (Fig 1).

### Stroop task

Over the 75 trials, the mean number of errors of all participants was very low (meditators: 0.39 errors, representing 0.52% of trials; novices: 0.49 errors, representing 0.65% of trials). The statistical analysis of the number of errors (Table 2) only revealed a significant effect of the task condition (*F*(1.65,65.87) = 16.18, *p*_*cor*_< 0.001, Ω² = 0.167, with more errors in the incongruent condition (0.75 ± 0.35 error) than in the neutral (0.33 ± 0.19 error, *p* < 0.001) and the congruent (0.20 ± 0.16 error, *p* < 0.001) ones, and that independent of the test and the group.

**Fig 2:**
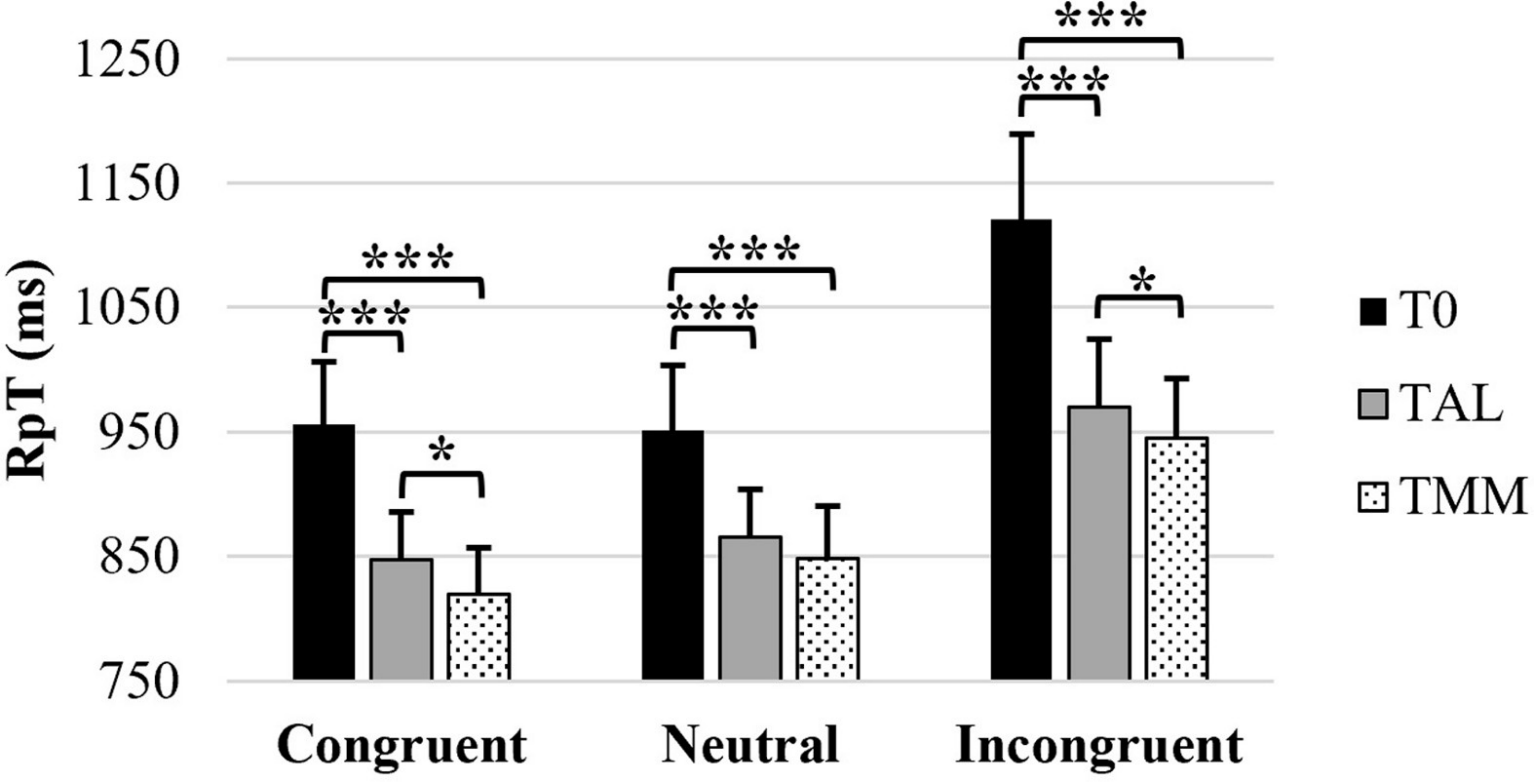
Stroop task mean reaction times (RT). Mean RT in milliseconds observed for successful trials for all participants (meditators and novices combined) across conditions. Error bars represent the normalized 95% confidence interval. Only the statistical significance of the decomposition of the condition × test interaction is represented. *p ≤ 0.05 and ***p ≤ 0.001.

For the RT of successful trials (S2 Fig), the ANOVA (Table 2 and Fig 2) revealed a significant effects of the task condition (*F*(1.6,64) = 100.47, *p*_cor_ < 0.001, Ω² = 0.151), test (*F*(1.48,59.12) = 52.92, *p*_cor_ < 0.001, Ω² = 0.137) and their interaction (*F*(4,160) = 5.83, *p* < 0.001, Ω² = 0.007). Post-hoc decomposition of the condition × test interaction showed that at T_0_, T_AL_, and T_MM_, RT_I_ was longer than RT_N_ (*p* < 0.001) and RT_C_ (*p* < 0.001), and at T_MM_, RT_N_ was longer than RT_C_ (*p* = 0.013). Furthermore, it showed that RT_C_, RT_N_ and RT_I_ were longer at T_0_ than at T_AL_ (*p* < 0.001) and at T_MM_ (*p* < 0.001). RT_C_, and RT_I_ were also longer at T_AL_ than at T_MM_ (RT_C_: *p* = 0.016; RT_I_: *p* = 0.030). RT values across all conditions, irrespective of meditation experience are presented in Table 3.

**Table 3:** Stroop task mean reaction times (RT) for all participants (meditators and novices combined) across conditions.

	T_0_	T_AL_	T_MM_	T_0_ + T_AL_ + T_MM_
	(M ± CI)	(M ± CI)	(M ± CI)	(M ± CI)
RT_C_	956.09 ± 50.59	847.62 ± 38.46	819.45 ± 37.06	874.39 ± 45.77
RT_N_	951.18 ± 52.07	865.51 ± 38.79	848.52 ± 42.41	888.40 ± 46.46
RT_I_	1120.50 ± 69.19	970.33 ± 54.25	944.96 ± 47.88	1011.93 ± 61.97

## Discussion

The present study investigated the acute cognitive effects of a mindfulness meditation session compared to an active control intervention, while controlling for possible cofounding factors related to the familiarity with this practice, the presence of an individual response, and the engagement in the intervention.

The participant’s engagement in both interventions was controlled using subjective questionnaires and HR recordings. We observed high scores (>7/10) in the post-MM self-assessment questionnaire in both meditators and novices, reflecting their ability to follow the given instructions during the MM session irrespective of their previous experience with this practice. The meditators scores were however higher than those of the novices (1/10 difference, large effect size). This suggests that a previous MM experience is associated with an improved (perceived) ability to engage in the proposed intervention. The verbal exchange following the control recording also revealed that both meditators and novices were attentive during the control intervention.

Irrespective of previous MM experience, the analysis of mean HR showed that both interventions slowed down the cardiovascular dynamics (lower HR, small effect size), but more so during the MM session. This latter finding is in accordance with previous studies [23, 24] meditate. It can be explained, at least partially, by the breathing exercise included in the MM intervention, which probably slowed the participants’ breathing and in return affected the cardiovascular dynamics [40]. Nevertheless, as a significant effect was also found during AL, focusing on the breath doesn’t seem the only mechanism mediating the observed slowing-down in HR. It could be also due to the quiescence state induced by the selective mental processing, combined, in the case of MM, to the diffuse attentiveness to the present experience, which helps in decreasing mental distress and developing a reflexive awareness [4].

The used subjective and objective measures suggested that all participants, independent of whether or not they had a previous experience in MM practice, were properly engaged in both interventions. We note however a divergence between the self-assessment score and mean HR, with the former showing a statistically significant group effect, but not the latter. It could be that HR is not after all an accurate objective measure of how successfully the participants meditated.

Cognitive performance was evaluated using the Stroop task. As a prerequisite, we first analyzed the number of errors which were overall negligible (less than 0.7% of the achieved trials). The number of errors was not affected by the intervention or the previous experience in MM. It was only affected by task condition, with the least errors occurring in the easiest congruent condition and the more errors in the most challenging incongruent condition (large size effect). These findings are not surprising as similar results were found in previous studies investigating the acute MM effects using the Stroop task [11, 14].

Regarding reaction times in Stroop task, our results showed that, compared to baseline, the participants were faster in the three conditions after both interventions (large size effect). They were additionally even faster after MM than after AL in the congruent and incongruent conditions, which supposedly reflects better selective attention, inhibition and cognitive flexibility abilities. Accordingly, our findings suggest that focusing attention on a specific object (the content of the recording in the AL, breath in the MM) may enhance the ability to select perceptual task-relevant information, and to inhibit stimuli that are not relevant to fulfill the task goal. In addition, it appears that orienting attention to the present experience, which was the specificity of the MM session, induced larger cognitive benefits. More importantly, our results suggest that both meditators and novices could show comparable acute cognitive benefits from the proposed interventions.

Due to the different ways in which the Stroop task has been implemented in previous studies (computer vs. paper, number of trials, absence or presence of neutral words, kind of neutral words) and analyzed (reported outcomes and their calculation), it is not straightforward to compare our results to those reported in the available literature. While some studies reported a lack acute effects on Stroop task performance following MM (e.g., [14]), others did report benefits (e.g., [22]). Anyhow, to our knowledge, no direct comparison was previously made between meditators and novices as in the present study. Here, we go even further by using a within-subject design. We also respected the post-intervention time delay that was shown to optimize the chances of observing acute effects [22]. Our results suggest that acute cognitive benefits of MM can be observed following even a single 10-minute session, without the need of a previous practice or familiarity with mindfulness training.

However, in the present study, the number of used cognitive tests had to be limited since acute effects are transient and their duration is still unknown. While we used a broad spectrum task (Stroop), the lack of specific tests to assert separately the implicated processes (attention, cognitive flexibility, inhibition) is a limitation. Another limitation of the present work is the absence of a passive control intervention in addition to the AL and MM interventions. As suggested by Davidson and Kaszniak [41], a rigorous approach must include several control groups designed to rule out all alternative explanatory mechanisms of the expected effects. Additionally, even if the intervention’s randomization permitted to exclude a potential test-retest effect, adding a second baseline test phase would further confirm the test-retest reliability of the used Stroop task and exclude any possible repetition effect that might contribute to the observed baseline vs. post-intervention differences. It must be acknowledged as well that some individuals could be disturbed by the laboratory setting, which could interfere with their ability to fully engage in the proposed intervention, and hence lead to an underestimation of the potential real-life benefits.

## Conclusions

The present study used a rigorously controlled within-subject design to investigate MM’s acute effects in comparison to an attentive listening intervention. It extends existing literature by showing that a single 10-minutes MM session can acutely enhance attention, inhibition and cognitive flexibility processes that are implicated in the used Stroop task, regardless of previous experience in mindfulness meditation practice. The observed cognitive benefits were also present, but to a lesser extent, after a simple attentive listening intervention. Hence, the MM component of orienting attention to the present moment seems to be crucial to optimize the cognitive benefits. These findings are encouraging and support the potential of both intervention approaches to enhance cognitive performance on the short-term, and presumably to improve brain functioning and well-being on the longer run, but this remains to be specifically addressed in future studies.

## Supporting information

S1 Fig Heart rate (HR) per group and intervention. Mean HR values ± normalized 95% confidence interval for meditators (A) and novices (B) at baseline and during the attentive listening (AL) and mindfulness meditation (MM) interventions.
(TIF)

S2 Fig Stroop task reaction times (RT) per group, condition and test. Mean RT values ± normalized 95% confidence interval in the three Stroop task conditions for meditators (A) and novices (B) at baseline and following the attentive listening (TAL) and the mindfulness meditation (TMM) interventions.
(TIF)

S1 File Instructions about the organization of the provided data files. (DOCX)

S2 File NN intervals data for the meditators group. (MAT)

S3 File NN intervals data for the novices group. (MAT)

S4 File Stroop task data for the meditators group. (MAT)

S5 File Stroop task data for the novices group. (MAT)
