# Supplementary figures and images for: A single session of mindfulness meditation may acutely enhance cognitive performance regardless of meditation experience
Source: PLoS One. 2023 Mar 15;18(3):e0282188. doi: 10.1371/journal.pone.0282188 (PMC10016675; doi:10.1371/journal.pone.0282188)

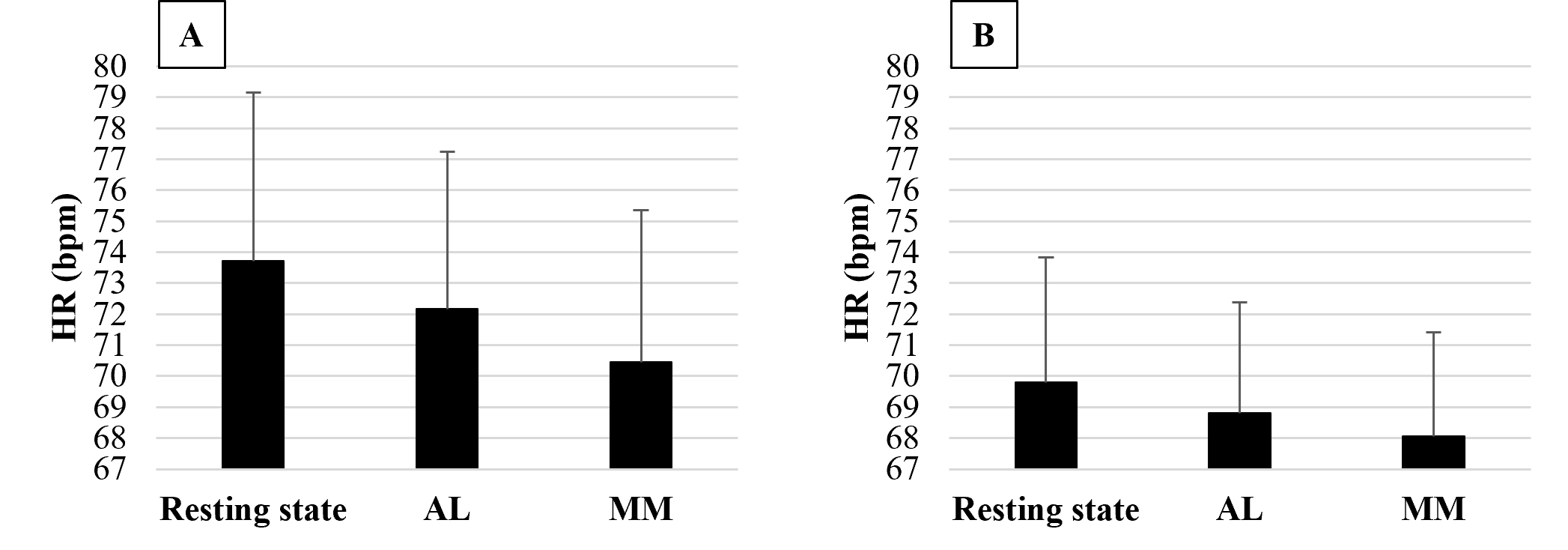

Supplement: S1 Fig — Mean HR values ± normalized 95% confidence interval for meditators (A) and novices (B) at baseline and during the attentive listening (AL) and mindfulness meditation (MM) interventions. (TIF) [file pone.0282188.s001.tif]

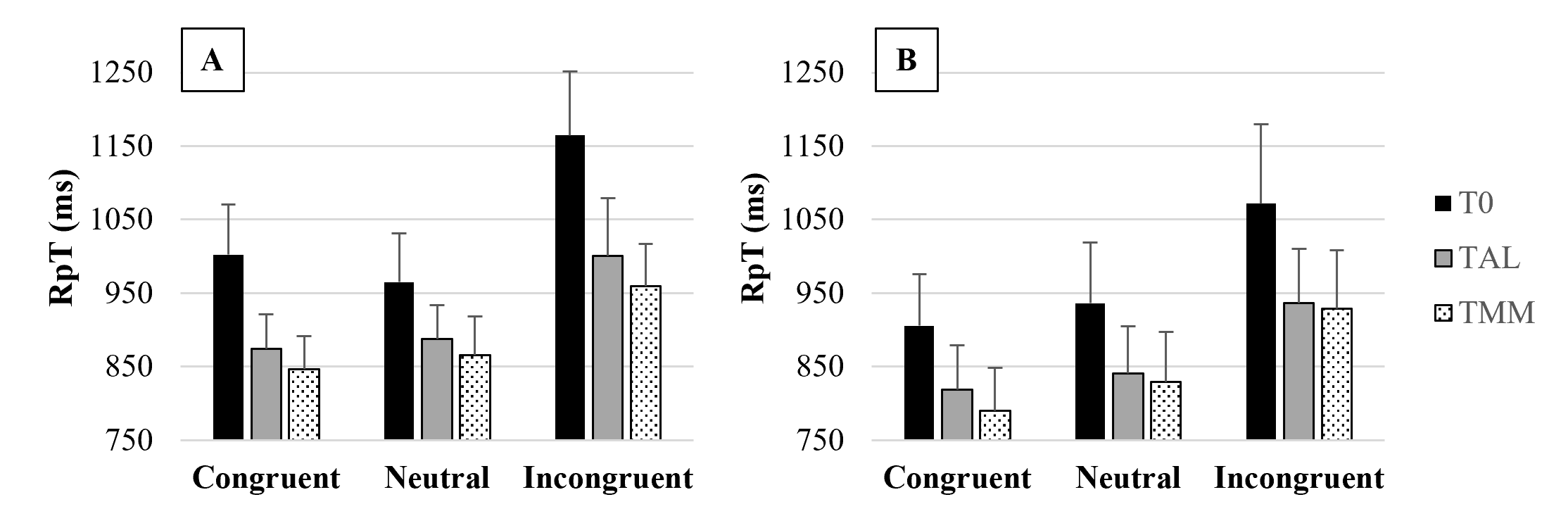

Supplement: S2 Fig — Mean RT values ± normalized 95% confidence interval in the three Stroop task conditions for meditators (A) and novices (B) at baseline and following the attentive listening (TAL) and the mindfulness meditation (TMM) interventions. (TIF) [file pone.0282188.s002.tif]
