# Supplementary material for: A single session of mindfulness meditation may acutely enhance cognitive performance regardless of meditation experience
Source: PLoS One. 2023 Mar 15;18(3):e0282188. doi: 10.1371/journal.pone.0282188 (PMC10016675; doi:10.1371/journal.pone.0282188)

**Data organization manual for data supporting information**

**Stroop data**

In the Matlab files (“*StroopMeditators*” or “*StroopNovices*”), there is one participant per line and one test per column (T_0_ is the first column, T_AL_ the second one, T_MM_ the third one).


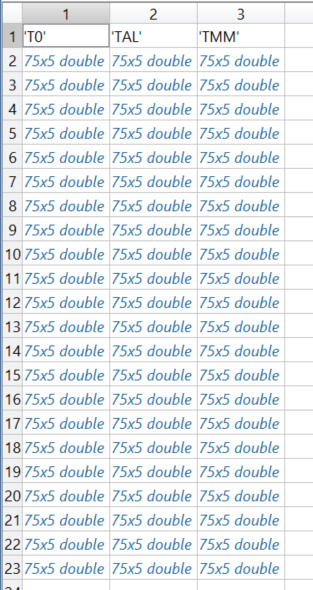


*One participant per line*

Each cell contains the **Stroop** **raw data** for the corresponding participant and test. There is one trial per line. The 5 columns correspond to the following:

- The first column represents the category of the displayed word: numbers 1 to 4 correspond to a color word (e.g., “rouge” meaning red), and numbers 5 to 8 correspond to a neutral word (e.g., “jambe” meaning leg).
- The second column represents the color of the displayed word.
- The third column represents the selected response key (if it’s the same number as in the second column, the participant would have answered correctly).
- The fourth column is the RT in seconds.
- The fifth column is a dichotomous code with 1 corresponding to a successful trial and 0 corresponding to a failed one.

To determine the word condition, you have to compare the first and second columns. If the number is equal in these two, the word was congruent. If not, the word was incongruent. If the number on the first column is higher than 4, the word was neutral whatever the number on the second column.

*Word’s category*

*Word’s color*

*Button pushed*

*Success/Failed*

*RT (s)*


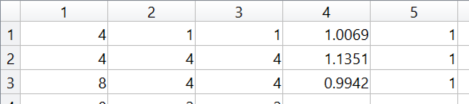


The participant gave the **right** answer.

*One trial per line*

The first trial is an **incongruent** one.

*(number ≤ 4 and first column ≠ second column)*

The third trial is a **neutral** one.

*(number > 4)*

The third trial is a **congruent** one.

*(First column = second column)*

**Polar data (HR)**

The supporting information files of the Polar include the preprocessed data.

In the Matlab files (“Polar*Meditators*” or “Polar*Novices*”), there is one participant per line and one intervention per column (resting state is the first column, AL the second one, MM the third one).


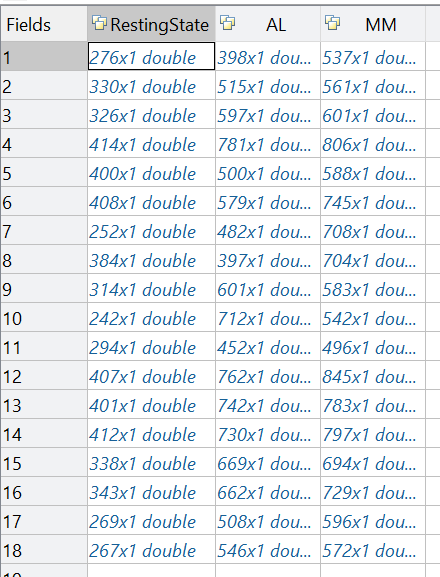


*One participant per line*

Each cell contain the **Polar preprocessed data** of the corresponding participant and intervention. There is only one column representing the time (in seconds) between two heart beats (NN interval).


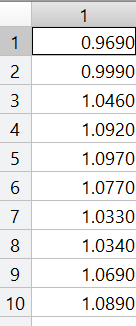

Supplement: S1 File — (DOCX) [file pone.0282188.s003.docx]
